# 2510 Disparities in navigation to health research among Floridians

**DOI:** 10.1017/cts.2018.66

**Published:** 2018-11-21

**Authors:** Linda B. Cottler, Deepthi S. Varma, Krishna Vaddiparti, Catherine Striley

**Affiliations:** 1 Department of Epidemiology, University of Florida; 2 Clinical and Translational Science Institute, University of Florida

## Abstract

OBJECTIVES/SPECIFIC AIMS: The analyses explore socio-demographic characteristics of community members who are navigated and enrolled in health research through HealthStreet—the CTSA community engagement initiative at University of Florida. METHODS/STUDY POPULATION: HealthStreet utilizes the Community Health Worker model to reach the community, conduct health assessments, provide referrals to medical/social services and link people to health research. We compared never navigated, navigated and not enrolled, navigated and enrolled on demographics, access to care, common health conditions and drug use among this community dwelling population. RESULTS/ANTICIPATED RESULTS: Among the 9581 community members, 51% were navigated to a study; 41% were screened eligible and enrolled (n=2024) for an overall enrollment yield of 21%. Disparities were found for all variables; never navigated Versus the others were more likely to be African American, never married, reporting less education and less access to care. The navigated and enrolled Versus others were older females who reported more education, food insecurity, more access to care, and higher rates of hypertension, depression, and prescription opioid and marijuana use. DISCUSSION/SIGNIFICANCE OF IMPACT: Our unique and comprehensive data can assist investigators to tailor recruitment efforts that reduce disparities in health research.

